# Role of Academics in Transferring Knowledge and Skills on Artificial Intelligence, Internet of Things and Edge Computing

**DOI:** 10.3390/s22072496

**Published:** 2022-03-24

**Authors:** Grzegorz Dec, Dorota Stadnicka, Łukasz Paśko, Maksymilian Mądziel, Roberto Figliè, Daniele Mazzei, Marios Tyrovolas, Chrysostomos Stylios, Joan Navarro, Xavier Solé-Beteta

**Affiliations:** 1 Faculty of Electrical and Computer Engineering, Rzeszów University of Technology, 35-959 Rzeszów, Poland; 2 Faculty of Mechanical Engineering and Aeronautics, Rzeszów University of Technology, 35-959 Rzeszów, Poland; dorota.stadnicka@prz.edu.pl (D.S.); lpasko@prz.edu.pl (Ł.P.); mmadziel@prz.edu.pl (M.M.); 3 Computer Science Department, University of Pisa, 56127 Pisa, Italy; r.figlie@studenti.unipi.it (R.F.); daniele.mazzei@unipi.it (D.M.); 4 Laboratory of Knowledge & Intelligent Computing, Department of Informatics and Telecommunications, University of Ioannina, GR-47150 Arta, Greece; tirovolas@kic.uoi.gr (M.T.); stylios@uoi.gr (C.S.); 5 Athena Research Center, Industrial Systems Institute, GR-26504 Patras, Greece; 6 Research Group in Internet Technologies & Storage, La Salle Campus Barcelona, Universitat Ramon Llull, 08022 Barcelona, Spain; joan.navarro@salle.url.edu (J.N.); xavier.sole@salle.url.edu (X.S.-B.)

**Keywords:** Internet of Things, Artificial Intelligence, Edge Computing, knowledge transfer

## Abstract

Universities play an essential role in preparing human resources for the industry of the future. By providing the proper knowledge, they can ensure that graduates will be able to adapt to the ever-changing industrial sector. However, to achieve this, the courses provided by academia must cover the current and future industrial needs by considering the trends in scientific research and emerging technologies such as Artificial Intelligence (AI), Internet of Things (IoT), and Edge Computing (EC). This work presents the survey results conducted among academics to assess the current state of university courses, regarding the level of knowledge and skills provided to students about the Internet of Things, Artificial Intelligence, and Edge Computing. The novelty of the work is that (a) the research was carried out in several European countries, (b) the current curricula of universities from different countries were analyzed, and (c) the results present the teachers’ perspective. To conduct the research, the analysis of the relevant literature took place initially to explore the issues of the presented subject, which will increasingly concern the industry in the near future. Based on the literature review results and analysis of the universities’ curricula involved in this study, a questionnaire was prepared and shared with academics. The outcomes of the analysis reveal the areas that require more attention from scholars and possibly modernization of curricula.

## 1. Introduction

Since the foundation of the University of Bologna, considered to be the first university in the world, in 1088 [1], educational institutions and advanced research centers [2] have played a key role in preparing gifted minds to meet the challenges of society. Such institutions have been referred [3] to as world knowledge repositories, sources of new discoveries that supply professional staff and engage in public debates. Communities of professors and researchers that belong to such institutions are considered [4] to be leaders in their fields of education, research, and technology, capable of preparing students for demanding positions both in academic and psychological terms.

Universities have traditionally assumed this enormous responsibility that society has placed on them. A prime example of the efforts made by academia and researchers to drive change is their mission to [5] achieve the United Nations’ Sustainable Development Goals (SDGs) [6]. The SDGs, which are a continuation of the Millennium Development Goals (MDGs) [7,8], address vital global challenges such as social cohesion, economic prosperity, and the protection of the environment to create a more sustainable planet and a brighter future for all. The SDGs define a framework [5,9] of common objectives, which can be divided into three general categories: (1) well-being, (2) environment, and (3) economy. In all three categories, universities are classified as institutions that play a fundamental role. In well-being, access to good quality education is considered [5,10] a crucial factor for sustainable development and a prerequisite for reaching the other objectives. Concerning the environment, universities are referred [5,11] to as institutions where vital research is carried out to enable us to understand natural phenomena and discover valuable knowledge from multiple fields and talents that will help us find solutions to the challenges of this constantly developing world. There is an increasing demand for highly qualified professionals (in digitalization, automation, and globalization) in the economy, and higher education [5,12] is regarded as a catalyst for innovation, as it facilitates international collaboration and contributes to more remarkable sustainable growth. Universities have become vital agents in any SDGs’ promotion and/or implementation initiative.

While universities continue to produce highly qualified, well-grounded graduates, and work to keep their study programs up-to-date, the Information and Communication Technologies (ICTs) sector keeps moving forward with several ground-breaking innovations and improvements which aim to make our day-to-day lives more manageable. In this way, the digitalization and exploitation of new technologies [13] have enabled us to transform traditional industries into smart factories and intelligent manufacturing environments [14]. Industry 4.0, or the fourth industrial revolution, offers new levels of organization along the entire value chain of the life cycle of products [15] which is itself making tremendous progress [16]. In this new industrial context [16], design principles such as decentralization, real-time capabilities, or virtualization, which go hand in hand with augmented and virtual reality, robotic automation and industrialization, big data analytics, cloud data, and computing or cybersecurity, have become the fundamental pillars of such a revolution. All of these factors contribute to the creation of an industry based on a set of interconnected systems that are capable of making decisions with little or no human intervention [17].

In this current industrial revolution, the three technological pillars are [18,19,20]: Internet of Things (IoT), Edge Computing (EC), and Artificial Intelligence (AI). Firstly, the Internet of Things provides us with the mechanisms needed to enable a dynamic, global network of heterogeneous objects based on standard communication protocols [14] that interact and cooperate to reach a common goal [21]. This multi-device integration and interconnection in a widely distributed network elevates the scope of traditional communication [22] from human-human to human-human, human-thing and thing-thing or Machine-to-Machine (M2M). Secondly, Edge Computing is a computational paradigm designed to split the computation process of the devices that are physically close to data generation points instead of sending the whole bulk of data to a remote entity. This makes it possible to address drawbacks such as latency, battery consumption, Wi-Fi availability, security, and privacy [23]. Finally, artificial intelligence facilitates automatic data analysis to extract knowledge models, a clear advantage in decision-making and optimization processes. The different AI methods analyze and extract actionable insights from all generated data [24], thus providing competitive advantages in manufacturing processes [25].

Industry 4.0, first named as such by the German government in 2011, is no longer a futuristic vision, but it has become a standard process for more and more industrial processes over time. The current industrial revolution is in full force, and there is an apparent demand for qualified professionals that are able and willing to respond to the needs and opportunities which arise in this new generation of industrial processes. However, do current university programs cover all the theoretical and practical aspects of Industry 4.0 to enable professionals to operate effectively in this paradigm? In the work [26], it was shown that technologies such as EC or IoT have a positive impact on the achievements of specific SDGs. Which areas (AI, IoT, EC) are more and which are less taught? What methods are used in teaching AI, IoT, and EC? How much do the real problems of the industry affect the educational teaching process? What are the academic needs in the field of AI, IoT, and EC and what could support the teaching process? Projects, practicals, and case studies should not only focus on current needs but also look to the future, considering current trends in research and taking advantage of market opportunities to solve real-life challenges (such as predictive maintenance [27]).

This paper aims to present a study carried out in a university educational community on how knowledge of IoT, EC, and AI is transferred in the teaching process and how students acquire such knowledge from the academic teachers’ point of view. To achieve this, a 7-stage methodology process is followed: (1) literature review, (2) university courses review, (3) defining the research questions, (4) questionnaire development, (5) survey, (6) survey results from quantitative and qualitative analysis, and (7) conclusions and development of recommendations. The review of the literature describes reaction of universities to the challenges of Industry 4.0. The university courses review contains information about actual training programs of selected universities in the field of AI, IoT, and EC. The questionnaires are prepared based on the extended Likert scale during the first phase. A series of multiple-choice questions with predefined answers is formulated. Once the questions have been determined, the survey phase is initiated, where a group of academic representatives distributed in over 17 countries participates. Once completed surveys have been collected, the results are analyzed to detect areas that require attention and possibly modernize the curricula. Finally, conclusions are drawn and recommendations are made based on the results obtained.

The main motivation that seeded this research comes from the document released on 14th June 2018 by the European AI Alliance [28]. This report discussed the competitiveness of the EU in Artificial Intelligence and declared that it is necessary to support the knowledge transfer between academic and productive sectors to enable a practical and widespread use of AI and Machine Learning (ML). From this document, it can be inferred that it is universities’ responsibility to (1) train a new generation of experts and (2) to increase the collective knowledge while sustaining the European educational offer by providing students and companies with training materials and strategies regularly updated. Additionally, according to the literature, the knowledge distribution of Industry 4.0 technologies (i.e., AI, IoT, and Edge Computing) around Europe is uneven [29,30,31,32]. This work aims to fight these circumstances by providing updated evidence of this situation nowadays in some regions of Europe and identifying those Industry 4.0 areas of the curricula that shall be upgraded.

The remainder of this paper is organized as follows. Section 2 presents the literature review in university training programs and Industry 4.0 digital technologies. Section 3 describes the research methodology followed to conduct the study. Section 4 discusses the results of the pre-empirical stage of the work. Section 5 shows the results of the study. Section 6 answers the research questions. Section 7 presents the recommendations derived from the analysis of the obtained results. Finally, Section 8 concludes the paper focusing on this work’s limitations and future research directions.

## 2. Literature Review

This section contains results of the literature review concerning response of universities to the issues related to the Industry 4.0 and digital technologies used in Industry 4.0.

### 2.1. Universities Response to Industry 4.0 Challenges

Industry 4.0 poses new challenges to university education and academics have responded with great interest by proposing applications and frameworks in which 4.0 can update the educational offer. Some works focus on the possibilities of a 4.0 transformation of university education itself, thus implementing an Industry 4.0-type experience in teaching activities or promoting innovation in the educational process. In this direction, [33] addresses the practical applications of Big Data analysis and IoT technologies to improve the delivery of higher education courses. Therefore, the work presents the use of 4.0 technologies to improve education (quality assurance purposes, data collection, a digital platform that uses these technologies, etc.).

Instead, other works focus on the new requirements or subjects to include in higher education curricula, perhaps providing frameworks or case studies. Götting et al. [29] regards the development of curricula within the 4.0 paradigm. Specifically, the work evaluates two study programs of the university of applied sciences Emden/Leer using the Reference Architecture Model for Industry 4.0 (RAMI4.0). The authors mainly focused on proposing this methodology to evaluate study programs concerning Industry 4.0. Another generic framework is proposed by Coşkun et al. In [34], presenting a framework for Industry 4.0 education to be applied at the Turkish German University of Istanbul. The framework considers three components (curriculum, laboratory, and student club) adapted to the 4.0 vision. The proposal for the curriculum component includes new study modules or changes to the existing modules. In [35], Mian et al. acknowledges the absence of design and implementation strategies in university education to adapt to the Industry 4.0 paradigm. Indeed few studies are addressing real scenarios that contemplate the possibility of updating course programs, especially from the point of view of their stakeholders (professors, researchers, and students). Therefore, the authors use a combined methodology of SWOT (Strengths, Weaknesses, Opportunities, Threats) and AHP (Analytics Hierarchy Process) analysis to investigate the stances of university stakeholders about the adaptation of university programs to Industry 4.0, focusing on how an Industry 4.0 transformation can be planned in universities: allocation of dedicated funds, staff training, but also updating curricula and industrial collaborations. A practical approach to upgrade the educational offer of universities within the 4.0 context is given by the work of Wanyama et al. [36] in which two sets of equipment for Industry 4.0 teaching support are presented, together with examples of project-based learning experiences.

There are also proposals for specific solutions adapted to fields and domains other than Engineering where Industry 4.0 poses new challenges, such as Life Sciences (food production, plant and animal sciences, etc.) and Tourism. The latter case concerns the use of 4.0 standard technologies (IoT, Big Data analysis, Virtual Reality) applied to the tourism sector. In [37], Bilotta et al. presents the course design and delivers results at the Course Degree in Tourism Science of the University of Calabria. The use of 4.0 technologies in Life Sciences is addressed by in [38] where is provided a general framework to support education in the adaptation to Industry 4.0 context and is presented the case study of an academic course design and customization at Wageningen University in the Netherlands.

Alongside these adoption proposals, it is possible to analyze the current status of the education sector linked to Industry 4.0 by observing the perceived needs or expectations of college students and what the universities offer them in their training process. In this direction, in [30] Oliveira and Sommer surveyed 733 college students from Brazil and Germany about their awareness of the current industrial context and their perception of preparedness. The study highlights a higher awareness about the topic Industry 4.0 and its related technologies in German students and a discrepancy between the perceived preparation in Industry 4.0 (I4.0) between first semester and last semester students (while in Brazil, there were no differences).

In [32], Paśko et al. surveyed 563 students mainly from European countries about their knowledge and skills in the area of IoT, AI, and EC. The authors concluded that students are familiar with AI technologies while they have a lack of education in the field of EC. Moreover, students have better theoretical knowledge compared to the skills of practical application of technologies in industry.

In [31], the authors present the results of a questionnaire-based survey developed to analyze the situation of three Italian universities regarding the readiness of its students to work in a 4.0 context. The analysis of the 433 questionnaires collected in the study emphasized the need for a broader knowledge of the foundations of Industry 4.0 and a possible revision of the courses that teach technical topics with the possibility of integrating the new teaching methodologies prompted by Education 4.0.

The investigation of the state of I4.0 readiness and awareness is also addressed in [39] where Tinmaz and Lee study this topic in the context of South Korean ICT students. Although the authors observe that the students interviewed in this study showed at least a limited knowledge about I4.0 and its implications, the results underline that a deeper understanding of this topics is needed, especially in finding the involvement and integration with the training activities in their curricula.

On the other hand, it may be necessary to understand the perceptions of the academics in implementing I4.0 in teaching and what students think about Industry 4.0 in the learning process. In [40] the authors performed qualitative research on 33 stakeholders involved in education to explore the perceptions of the teaching staff about the I4.0 readiness of the education sector (meaning not only universities) in South Africa. Oke and Fernandes found a general lack of exploitation of 4.0 technologies in teaching and learning processes and that the current curricula do not provide the skills required for I4.0.

The fourth industrial revolution demands transformation in engineering curricula structure to provide the future workforce with the skills required to operate in this new environment. Within this topic, Ref. [41] examines what the possible implications of I4.0 are in the development of university programs in South Africa. Through a literature review, the analysis of industrial engineering curricula and a questionnaire-based survey conducted among 10 universities, the authors observed that: universities need a better alignment with I4.0 and increase the interdisciplinarity of their courses; the principles of I4.0 should be included more in teaching modules and the material in order to meet companies’ needs in terms of required skill set.

The need for change invests in the topics covered in university courses and the teaching approach itself. In [42], the authors employ the systematic literature review method to investigate the current state of engineering education in the I4.0 context, particularly concerning additive manufacturing. Motyl and Filippi note that more active teaching involving direct participation (such as in project-based learning) is necessary; nonetheless, not always well welcomed by the teaching staff.

As observed in the literature, there is a high interest in Industry 4.0 challenges in the academic world. However, many studies on Industry 4.0 readiness from a higher education perspective come from non-EU countries [34,35,39,40,41]. Those assessing in Europe the level of preparedness of students for I4.0 may need a broader European view, as they focus on individual countries such as Germany [29,30] and Italy [31] or cover only a few European countries [32]. Moreover, such studies may need updating since, in the meantime, the requested skills and university programs may have been changed. Finally, survey-based studies have been carried out primarily on students [30,31,32,39], often leaving out the role of teachers and instructors (with the exceptions of [35,40]) and what they think of the current courses they teach, and if these courses are providing a good 4.0 training to their students that they can become the next workforce in the industry. Therefore, in this study, we propose a perspective on the current state of European universities courses related to the I4.0 context and its technologies and how teachers think university courses need to be upgraded.

### 2.2. Technologies Related to AI, IoT, and EC Required in Industry 4.0

To determine which technologies related to AI, IoT, and EC are required by the Industry 4.0 authors of this work used review articles. Xu et al. in [43], Peres et al. in [44], and Mabkhot et al. in [26] identified key technologies for the Industry 4.0: Industrial Internet of Things, Big Data and analytics, cloud computing, simulations, augmented reality, additive manufacturing, horizontal and vertical system integration, autonomous robots, cybersecurity. Zheng et al. in [45] presented a review of digital technologies in industry 4.0 applied in the manufacturing context. They concluded that blockchain is not as widely discussed in the domain of I4.0. This issue was targeted in [46] where Moosavi et al. conducted a systematic review through bibliometric and network analysis to show how blockchain can improve supply chain management. Additionally, the review of the newest application works indicates that technologies such as fuzzy systems, optimization or IoT are find practical in particular fields of Industry 4.0: Fallahpour et al. in [47] shows an application of a fuzzy inference system in supply chain management, Yuan et al. in [48] presents solving a scheduling problems with the use of a fruit fly optimization algorithm, and work of Salehi-Amiri et al. [49] contains an example of combining optimization algorithms with applications of IoT-based devices to improve a waste management system.

## 3. Work Methodology

This section presents the research methodology. The general scheme of the procedure is discussed as well as the literature and universities curricula review plan, rules of the research questions definition, principles of building the survey and sample selection criteria, and the survey results analysis plan.

### 3.1. General Description of the Methodology

**Figure 1:**
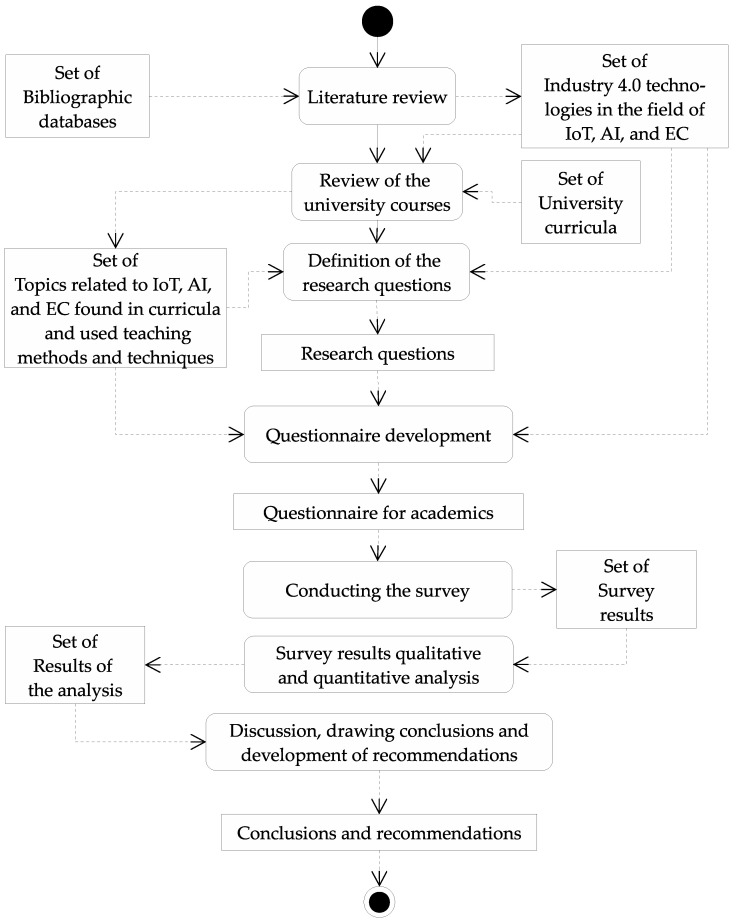
UML activity diagram of work methodology.

The research methodology schema expressed as the activity diagram of the UML 1.5 [50,51] is shown in the Figure 1. The methodology is an adaptation of a standard empirical research model that is described in the literature [52].

1. Literature review of universities’ response to industry 4.0 challenges and technologies used by the industry 4.0.
2. Training programs review to identify content dedicated to AI, IoT, EC.
3. Definition of the research questions.
4. Designing the questionnaire for academics to collect information about the actual state of teaching AI, EC, IoT.
5. Conducting the survey among university teachers.
6. Analysis of data collected in surveys.
7. Drawing conclusions about academics’ needs and recommendations on training programs changes.

### 3.2. Literature Review Methodology

The analysis and review of the State of the Art were done utilizing research papers dealing with the reception of Industry 4.0 in higher education. For this reason, databases such as Google Scholar, Scopus and ScienceDirect were utilized, focusing on the following keywords: “Industry 4.0” AND “education” OR “university”; to narrow the search was later added: “survey” OR “questionnaire” AND “curriculum”.

The second literature review process was to identify these topics of AI, IoT, and EC, which are used by Industry 4.0. The mentioned earlier bibliographic databases were queried for the keywords: “systematic literature review” AND “Industry 4.0” AND “IoT” AND “EC” AND “AI”. Then selected works from 2021 and 2022 were reviewed in terms of practical applications of digital technologies in Industry 4.0.

### 3.3. Review of Selected Educational Programs—Methodology

The assessment of the actual state of university training programs can lead to the consideration of how those training programs can be further developed according to what academics find helpful in properly training students. The source of data for training programs were universities belonging to the PLANET4 project consortium [53]: Università di Pisa (Italy), Universitat Ramon Llull (Spain), University of Ioannina (Greece), Rzeszów University of Technology (Poland). The review of training programs of these schools was performed in November 2020. For data collection, the following structure was designed:

- university name,
- program name,
- specialization (optional),
- semester,
- course name,
- BSc/MSc,
- module name,
- area: one of AI and ML, IIoT, EC,
- intended learning outcomes (ILO) connected with AI and ML, IIoT, EC,
- teaching and learning activities connected with AI and ML, IIoT, EC,
- used software and infrastructure,
- teaching methods and techniques.

The output data collection contains selected records. The selection criteria were as follows: the course description must contain topics from the field of AI, IoT, and EC, which are used in Industry 4.0. The required technologies were identified based on a literature review (see Section 4.1). The results of the universities curricula review are shown in the Section 4.2.

### 3.4. Definition of the Research Questions

The analysis of the curricula and the literature review results showed that the following areas could be explored:

- the presence of real industrial problems in the curricula,
- level of teaching topics related to AI, IoT, EC,
- level of the application of various development tools in the teaching process,
- teaching methods,
- difficulties in the teaching process.

### 3.5. Development of the Questionnaire

The questionnaire developed and used in the research consists of open questions and questions with predefined answers based on an extended Likert scale [54] with the following answers: not at all, to a small extent, to some extent, to a moderate extent, to a great extent, and to a very great extent. An atypical six-point scale was used in the research. The answer “not at all” has been added to a five-point scale to clearly highlight those topics that are not covered at all in the educational process. Thus, the median value (“to a moderate extent”) has shifted to the right. This may have led the respondents to find this answer positive rather than mediocre. The results were analyzed, taking this into account. The questionnaire aimed to assess to what extent Industry 4.0 topics are covered in EU higher education (HE). The following explanation was added to the answers to help respondents choose the proper answer:

1. To a small extent—Theory only
2. To some extent—Theory and exercises
3. To a moderate extent—Theory and practical examples
4. To a great extent—Theory and practical example based on real data
5. To a very great extent—Theory and practical application

The structure of the questionnaire is as follows:

- General questions covering the country (or countries) where the teacher is teaching and his/her experience,
- AI-related questions,
- IoT-related questions,
- EC-related questions.

Questions in the studied areas related to:

- Topics,
- Tools and software/environment,
- Applications,
- Learning techniques,
- Difficulties in teaching,
- Needs.

The received answers were analyzed to answer the Research Questions (RQs).

### 3.6. The Sample Selection Method and Survey Plan

As the qualitative and quantitative analyzes were planned, the minimum sample size was chosen as 40 according to the literature recommendations [55,56]. The authors planned to conduct the survey among 30 academics from the countries of universities included in the Planet4 consortium, and among 10 academics from other countries. The first version of the survey was put to a test trial. Subsequently, adjustments were made, and the final version of the survey was published. The survey contained closed-ended questions presented in Tables 4–27, questions about country of the participant, field of teaching, teaching experience, and open-ended questions about software used in teaching AI, types of projects related to EC, and difficulties in teaching.

### 3.7. Results Qualitative and Quantitative Analysis—Methodology

The received answers were reviewed in the first step to check their quality. Next, the quantitative and qualitative analyses were conducted. First, a general analysis of the obtained results was carried out, taking into account all three areas of research: Artificial Intelligence (AI), Internet of Things (IoT), and Edge Computing (EC). Then, each research area was analyzed individually. Only data from questionnaires where interviewees confirmed that they teach a course with contents related to AI, IoT, or EC were considered.

Qualitative analysis was performed to answer questions about theoretical and practical issues related to AI, IoT, and EC in university training programs.

Quantitative analysis was performed to investigate the degree to which the teaching topics are related to theory, data processing techniques, programming tools, and practical applications of AI, IoT, and EC.

## 4. Results of the Information Sources Review

The following subsections contain the results of the pre-empirical stage of the work. The summary of the literature review as well as details of courses review and research questions are presented.

### 4.1. State of the Art Analysis Results

Thanks to a thematic analysis of the appropriate found papers already presented in the literature review, the following conclusions can be drawn: researches concern countries outside the EU or describe the internal situation in selected EU countries; it is worthwhile to update researches due to changes in the university curricula; the point of view of academics should be more taken into account. Detailed results of the literature review were presented in the Section 2.1.

The papers [26,43,44,45,46,47,48,49] were selected as the source of information about these technologies related to AI, IoT, and EC, which are required by the Industry 4.0. The Section 2.2 details the results.

### 4.2. Courses Review Results

**Figure 2:**
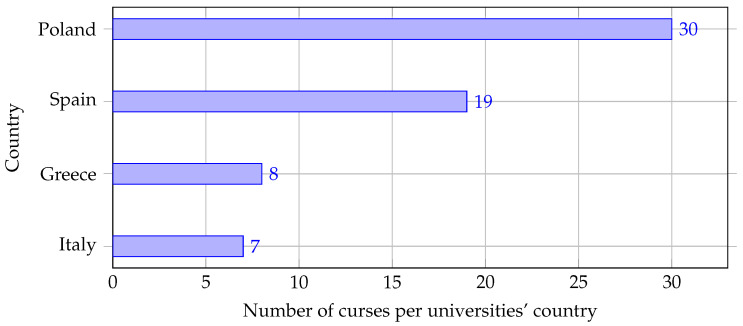
Number of courses per universities’ country.

In this section the results of the courses review are presented. Figure 2 shows the number of courses for each country.

The courses are part of twenty-six bachelor’s degree and thirty-eight master’s degree programs in which partners and associate partners from the PLANET4 consortium are involved, for a total of sixty-four courses. As these data come from several countries worldwide, it could be considered a significant sample to infer the current situation of university training programs.

**Figure 3:**
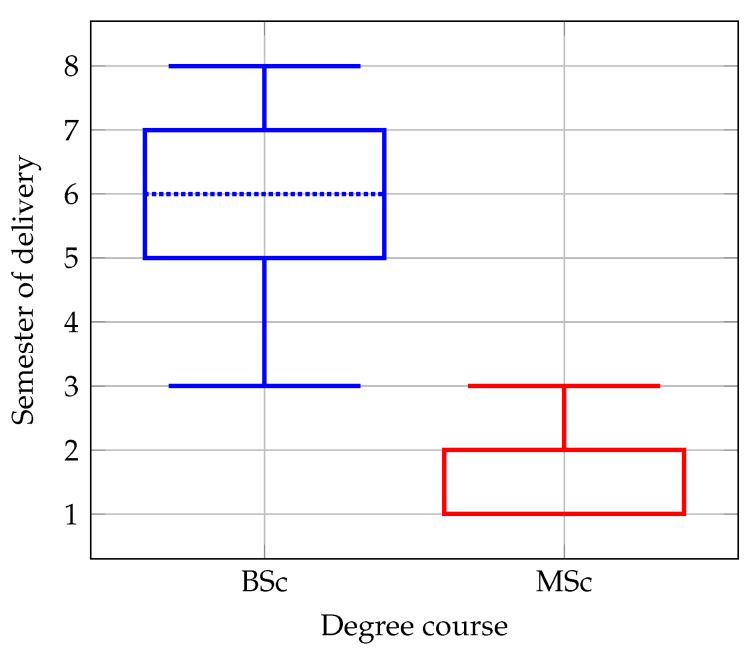
Semesters in which the courses are delivered.

The semesters in which the AI, IoT, and EC courses are delivered in bachelor’s and master’s degrees are both presented in Figure 3. The data collected shows a large difference in the semester of delivery of the courses related to Industry 4.0 between bachelor’s degree (median = 6, minimum = 3, maximum = 8, first quartile = 5, third quartile = 7) and master’s degree (median = 1, minimum = 1, maximum = 3, first quartile = 1, third quartile = 2). Since the bachelor’s degree in all the countries of the selected universities lasts three to four years, it can be observed that all the courses are preferably delivered at the end of the degree program, whereas in the master’s degree this type of courses are offered in the beginning of the degree programme.

**Figure 4:**
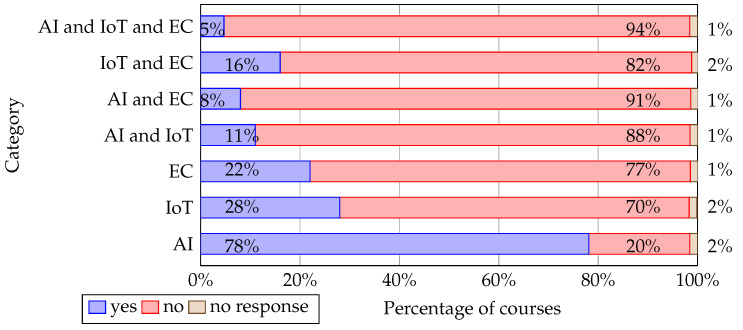
Percent of university AI, IoT, and EC training programs (total 64).

The number of AI, IoT, and EC university training programs analyzed is presented in Figure 4. The most taught discipline is AI (78% of courses), where IIoT and EC are less taught. Observing how the disciplines are taught together, IIoT and EC are frequently addressed in courses where another discipline is delivered.

The main programming languages for AI, IoT, and EC courses are presented in Table 1. In the AI area, the selected courses use more Python, Matlab and R programming languages, while in the IoT area, R programming language is used more often and in the EC area the courses use mainly Python programming language.

The main software applications, environment, or services used in the analyzed courses are presented in Table 2. The main environment used in the AI area courses is Matlab, but there is also significant variability in the software used. There is little evidence to show a preference in environment choice in the IoT and EC area courses, although Azure services and Arduino SDK are mainly used. Teaching methodologies applied in the selected training are presented in Table 3.

### 4.3. Research Questions

The main goal of the work was to evaluate how the knowledge from the field of AI, IoT, and EC required by Industry 4.0 is transferred to students during the university teaching process. For this work, the following research questions were defined:

**RQ1:**  Which areas (AI, IoT, EC) are more and which are less taught?
**RQ2:**  What topics and tools in the field of AI are taught and at what level?
**RQ3:**  What topics and tools in the field of IoT are taught and at what level?
**RQ4:**  What topics and tools in the field of EC are taught and at what level?
**RQ5:**  What methods are used in the teaching process related to the investigated area?
**RQ6:**  How much are the real industrial problems met in the teaching process?
**RQ7:**  What are the academic needs in the field of AI. IoT and EC and what could support the teaching process?

The phrased research questions enable us to assess what and how is taught in the analyzed areas. Additionally, it was important for the authors whether the teaching process includes references to real industrial problems that can be solved using AI, IoT, and EC technologies.

## 5. Results of the Survey

This section presents the results of the data collection process. First, we show a general description of the survey participants. The following subsections contain answers to the questions related to AI, IoT, and EC.

### 5.1. Survey Demographics

**Figure 5:**
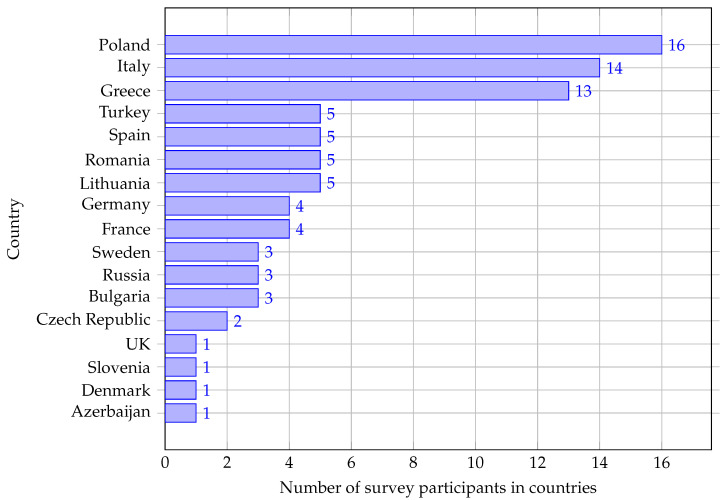
The number of academics from countries surveyed.

The questionnaire was distributed to the academics using two channels: named invitations sent to selected persons and public announcements on the Internet requesting to fill in the survey. One hundred forty-four (i.e., 144) persons filled in the questionnaire. Fifty-eight participants answered that they do not teach AI nor IoT or EC. 86 responses were used in further analyzes. They represent 17 countries (Figure 5). The higher number of academics represents Poland (16), Italy (14), and Greece (13).

### 5.2. General Overview

**Figure 6:**
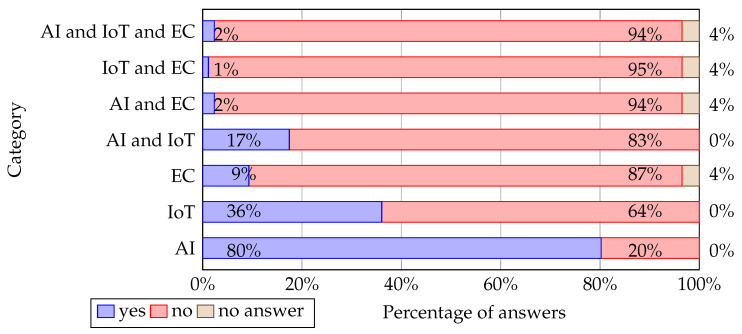
Percent of academics teaching AI, IoT, and EC (86 answers in total).

**Figure 7:**
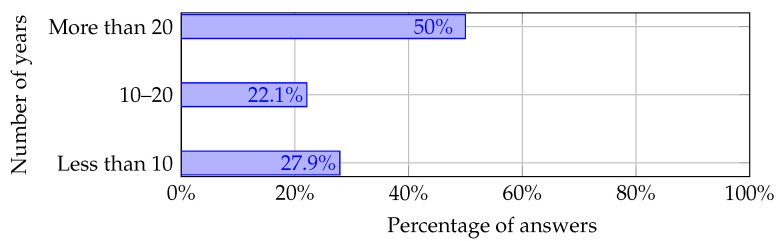
Academics’ teaching experience (86 answers in total).

The number of academics teaching AI, IoT, and EC is presented in Figure 6. Regarding the teaching of edge computing, three academics did not answer. Most of them (80% of respondents) teach artificial intelligence, while edge computing is the least taught research discipline. The teaching experience of the questionnaire respondents is shown in Figure 7.

About 50% of academics teach more than 20 years. The youngest placement persons who teach less than 10 years make up 27.9% of all answers; 22.1% of answers belong to the third category—teaching experience from 10 to 20 years.

### 5.3. Results Related to AI

Table 4 contains the answers to the question about disciplines used in teaching AI. The most used disciplines are computer science, mathematics and logic, while the philosophy of mind and linguistics are the least used disciplines.

Table 5 presents information about areas of AI that are taught by academics. The most commonly taught areas are machine learning, deep learning, data mining, and computational intelligence, while the least common are cognitive computing, natural language processing, and computer vision.

Table 6 shows answers to the question about teaching machine learning techniques used in artificial intelligence. Supervised and unsupervised learning techniques are taught at most, while reinforcement learning and semi-supervised learning are not popular.

Information about teaching deep learning models is presented in Table 7. Collected data show that models contained in the question are not taught often. The most popular is the convolution neural network which has 40% answers pointing out a small contribution in teaching. This characteristic is even higher on the other models, omitted from the teaching process of 45% up to 67% of academics. Answers may indicate that academics are teaching other models than those presented in the questionnaire.

Table 8 presents answers to the question about teaching the phases of the data mining process. Business understanding and deployment are skipped in the teaching process as arises from the answers. The first mentioned phases are related to general business management, while data mining is more bound to computer science. Academics who come from IT disciplines may not be management experts. Deployment of data mining solutions in a business requires conducting specific R&D work, which may be why this topic is not popular in teaching.

Table 9 contains answers to the question concerning three main techniques of computational intelligence (CI). Data show that teaching of CI focuses mainly on neural networks. Fuzzy systems and genetic algorithms make a more negligible contribution to teaching programs.

Data from Table 10 are consistent with Table 5. Academics attach little importance to natural language processing (NLP), so NLP techniques are taught to a small extent.

According to Table 5, computer vision and cognitive computing are faintly present in teaching. So the techniques used in these areas of AI are also seldom discussed, as shown in Table 11 and Table 12. Some methods of computer vision appear in teaching.

Data contained in Table 13 concludes that the most popular programming languages for AI applications are high-level programming languages: Python and Matlab.

Other mentioned programming languages used in AI teaching are: (1) mentioned once—Agent-Oriented programming languages (AgentSpeak, DALI), Julia, CLIPS, IDEF, UML, Scala, SQL, Kotlin (Android); (2) mentioned twice—C#, Javascript.

Table 14 shows that academics use no universal software package to teach AI. Other mentioned programming languages are: Apache Spark, BPMN, DTREG Predictive Modeling Software, EXSYS Professional, Google Colaboratory, IBM SPSS Modeler, Infor Coleman, Intellij IDE, Jade, Jupyter, Keras, Knowledge Extraction based on Evolutionary Learning, Minitab, Natural Language Toolkit, Netbeans, Node.js, NumPy, NY Dataset, Pandas, PostgreSQL, Protégé, Pytorch, Rapidminer, Robot Operating System, Scikit-learn, Self-written programs and algorithms, Sicstus Prolog, Spade, Tensorflow, Unity, Visual Studio, Waikato Environment for Knowledge Analysis, YOLO: Real-Time Object Detection. We can see that teachers work with standard programming environments (IntelliJ, Netbeans), specialized AI systems (YOLO, IBM SPSS Modeler, Tensorflow), or write their software. Teachers use varieties of programming languages and environments. According to Table 14, MS Excel is a widely used tool.

Optimization, computer vision, and anomaly detection are the leading AI applications that are taught by academics, as shown in Table 15. Analyzing the mentioned table, we can say that the least discussed issues are deliveries, cognitive systems, and supply chain management. Additional listed AI applications are as follows:

- Semantic web search,
- Unity,
- Smart environments,
- Cooperation and Negotiation among agents in Multi-Agent Systems,
- Churn prediction, business management,
- Text mining, data mining, machine translation, AI in humanities,
- Image translation, uncertainty modeling, manifold modeling, ensembles of NN, Bayesian networks etc.,
- Biometric person identification, viral evolution prediction, remote sensing,
- Numerous visual online demonstrators of methods,
- Medical diagnosis,
- Rule-based expert systems,
- Programming robots,
- Medical imaging, satellite imaging,
- Computer Aided Process Planning in Machining,
- Human-computer interaction, gesture and sign language recognition,
- IoT,
- Model-based diagnosis, Personal assistants,
- Modern Control Theory and Applications,
- Energy Systems,
- General Problem Solving,
- Arduino IDE.

Some answers can be classified as a computer vision application (medical and satellite imaging, gesture and sign language recognition, image translation).

Table 16 presents learning techniques used in teaching AI. The primary learning technique is a lecture, while problem-based learning and workshops are seldom used. E-learning seems to be also quite popular. The reason could be the lockdown caused by the COVID-19 pandemic.

### 5.4. Results Related to Internet of Things

This section contains the questions and the participants’ answers regarding Internet of Things; 31 academics declared teaching IoT, which is 45% of the number of persons devoted to AI. Fifteen teachers claim to be professionals in both AI and IoT (17% of the questionnaire participants). Moreover, only two are experts in all of the three examined domains.

The goal of the first queries is to investigate which areas of IoT are the subject of teaching (Table 17). Collected data show that too little effort is made to teach security problems (searching for vulnerabilities, basic network attacks, cybersecurity).

Table 18 contains answers to questions about teaching context IoT. Results point to the fact that teachers could pay more attention to the issues concerning market behavior and deliveries.

Data presented in Table 19 shows how academics teach the application of IoT in different technologies. The conclusion arising from these data is that more pressure should be put on teaching topics concerning the deployment of IoT products and technologies in the production environment (containers, SaaS, PaaS, IaaS).

Table 20 presents learning techniques used in teaching IoT. The primary learning technique is the lecture, while problem based-learning and workshops are seldom used. E-learning also seems to be quite popular. The reason could be the lockdown caused by the COVID-19 pandemic.

Arduino is the most popular platform used in IoT teaching, as shown in Table 21. Academics also teach applications of cloud services in the IoT area.

The high level of answers “not at all” to the question of application of Map Reduce model denotes that IoT is probably treated as a technology for developing intelligent sensors and processing data gathered by these sensors, and maybe, for this reason, it is not in the teaching program of interviewed academics.

### 5.5. Results Related to EC

Only eight persons replied to the questionnaire concerning edge computing. Therefore, it is difficult to draw reliable conclusions from such a small number of responses. Therefore, we will only present the responses obtained.

Table 22 informs that just like in the case of IoT, privacy and security are the topics that could be more present in the study courses.

Table 23 presents an overview of technologies used in the EC implementations. Other mentioned technologies are as follows: AWS EC2 (low level), Azure (low level), Hadoop (low level), Smart TV, and Playstation 5 Edge Computing. Academics teach these technologies equally.

Table 24 presents algorithms and techniques. They are taught to an equal degree.

Academics claim that students have practical skills in edge computing (see Table 25). They do not use hardware/software platforms shown in Table 26. The EC topics are related to the IoT. A popular IoT platform is Arduino (see Table 21), so it is possible that students in edge computing projects also use Arduino. Other software/environments indicated in the questionnaires as used in teaching. Edge Computing is Mobile Edge Computing and Docker.

Data shown in the Table 27 suggests that the edge computing issues are taught in various applications. Moreover, teachers admit that they are implemented the following projects related to edge computing:

- Conversational Agent,
- Supervised Electronic Exam System, which focuses on identity recognition and exam security and starts in smartphones before clouding the data,
- Network optimization (in the context of performance and energy), mobile edge computing,
- E-health,
- Video streaming projects,
- Resilience in smart cities,
- Intelligent sensor systems in geosciences & health areas,
- FPGA based devices for intelligent signal processing.

Teachers indicated the most difficult in teaching Edge Computing are:

- Different Ecosystems.
- The various specification and resolutions of smartphones and internet connection.
- Motivating students for designing and implementing even such smaller models.
- Designing significant lab activities.
- Accessing to available technology freely.
- Deployment,
- Students tend to have a poor background in hardware design.

Answering the question about what may facilitate the teaching process of Edge Computing, the teachers answered:

- Both, internship and joint projects could be effective to motivate students.
- Maybe limited but free access to well-known platforms may facilitate the teaching process of Edge Computing.
- Joint projects with other institutions in academia or industry. Having free licensing for students.
- Industrial use cases.

Answers to questions concerning difficulties and potential facilitations point to the fact that edge computing is a new discipline and academics need cooperation with industry to develop suitable study courses.

To summarize, it needs to be underlined that the presented analysis is performed based on only eight answers coming from academics who teach Edge Computing, while 75 teachers answered that they do not teach Edge Computing.

## 6. Discussion

This section provides an overview of the research results and answers to the research questions.

**Figure 8:**
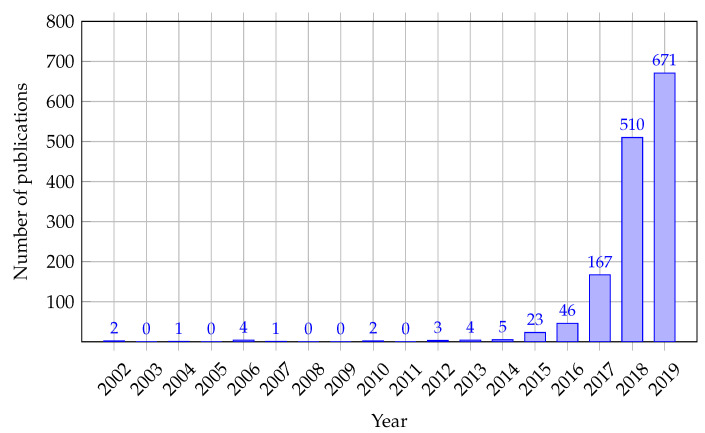
Number of publications per year concerning EC (based on data from [57]).

Regarding the first research question (**RQ1**), from the presented survey results (see Figure 6), it can be concluded that AI is the most taught. In 80% of questionnaires, teachers indicated that they teach issues related to AI; 36% of teachers indicated that they teach IoT, and only 9% of teachers indicated that they teach EC. The reason why so few teachers teach EC-related topics may be that edge computing is a new domain of science, as Figure 8 shows (see [57]). Therefore, academics still have not adapted learning programs to the new knowledge.

Regarding the second research question (**RQ2**) the research results (see Figure 6) showed that 80% of academics who participated have included AI teaching in their courses, resulting in this scientific field being much more widespread in curricula than IoT and EC. This is also evidenced by the fact that 78% of the total courses are AI-related (Figure 4). The teaching process of AI is based mainly on concepts and principles of computer science, mathematics, and logic (Table 4). In addition, most taught AI subsets are machine learning (mainly supervised and unsupervised learning), deep learning, data mining, and computational intelligence (Table 5). Furthermore, in improving students’ practical skills, the most taught programming languages for AI solutions deployment are Python and Matlab (Table 13). At the same time, more attention has been paid to optimization problems, computer vision, and anomaly detection for application areas (Table 15).

We have compared the results which we have obtained with other published works. For example, the authors of [58] investigated the courses offered at the undergraduate and postgraduate level by highly-rated schools such as Carnegie Mellon University, Massachusetts Institute of Technology (MIT), and University of California– Berkeley. According to this study, these universities teach an average of 10 AI-related courses, with a greater emphasis on machine learning (three courses per university), computer vision (two courses per university), natural language processing (two courses per university), and robotics (two courses per university). At the same time, each school offers at least one introductory AI course and advanced courses like knowledge representation and automated reasoning. However, contrary to the results of our questionnaire, deep learning is only offered at seven of the 20 top-ranked schools, with the rest having included some deep learning topics in their machine learning lectures.

A similar study was conducted by Cadoli and Carizzi, who did a questionnaire-based survey about teaching AI in Italian universities [59]. Collecting data from members of the Italian Association for Artificial Intelligence (AI * IA) and colleagues, the authors investigated the number of AI courses taught in Italian higher education, the number of students participating in them, and the educational level we encounter most of them. According to the paper results, AI courses are offered at 19 of the 80 universities, with 73 courses total. The 42 courses deal exclusively with AI topics and applications, while the remaining 31 contain some relevant sections (to be precise, AI topics cover half of the syllabus in these courses). Moreover, AI lectures are found at all higher education (HE) levels (undergraduate, Master, and Ph.D.), with most of them taught in the postgraduate program. Universities in Italy focus mainly on the basics of Artificial Intelligence, robotics, automated machine learning, automated reasoning, and knowledge engineering. As it is presented in [59], 3000 Italian students attend AI-related courses each year. This may be due to recent advances in artificial intelligence, which have influenced student participation in such courses.

The thorough search of the relevant literature yielded one more comprehensive research work in which the Brazilian public universities’ approach in AI and its subjects was investigated [60]. Explicitly analyzing the curriculum of 25 universities, the number of courses related to the AI knowledge area was recorded (20 required and 30 elective courses). At the same time, the topics to which more attention is given were identified. As stated by the authors, machine learning, heuristic search, neural networks, and knowledge-based/expert systems are the most popular research areas, unlike computer vision, where it is not mentioned anywhere. Several professors also mention fuzzy logic without however much deepening being done. Finally, an essential piece of information extracted from this study is that most professors have difficulty finding practical examples, probably due to the still little practical use of AI in industry or because enterprises are reluctant to share information about AI applications. Hence, students do not learn enough techniques and methods to implement AI applications, which is a significant barrier to implementing Industry 4.0.

The results of the research were able to give us an answer to the third research question (**RQ3**). Specifically, in the case of IoT, according to the questionnaire results presented in Figure 6, we can observe fewer academic professionals than AI (36% of the survey respondents is devoted to IoT, compared to 80% AI teachers). In addition to this, as shown in Table 17, most curricula include IoT fundamentals and application scenarios, where IoT technologies are employed without referring extensively to industrial case studies. From the same table, we can conclude that teaching programs could contain more security issues, while more emphasis could be placed on market behavior and deliveries (Table 18). In addition, according to Table 19, universities should focus more on subjects concerning the deployment of IoT products and technologies in the production environment (containers, SaaS, PaaS, IaaS).

These results largely agree with similar surveys that have been conducted. Similar research results were presented in [61], where the authors explored the IoT curriculum offered by five out of the top public and private Malaysian universities. According to their results, only the top two private universities (Asia Pacific University of Technology and Innovation (APU) and INTI University) have included IoT courses in their undergraduate programs. INTI International University offers a Bachelor (BSc) of Computer Science (Hons) major in Cloud Computing, which deepens in areas such as machine-to-machine (M2M) communications, Cloud Computing and Virtualization, Routing Protocols and Concepts, Cloud Security, and others. On the other hand, APU also offers BSc (Hons) in Information Technology, where the fundamentals of IoT such as networking, wireless sensors, architectures, and network protocols have been integrated along with concepts like localization and routing, and energy harvesting are taught.

Other researchers in China have concluded similar analyzes and results. Du, et al. [62], investigates the undergraduate curricula in IoT Engineering in China. As reported in this article, since 2011, almost all Chinese universities have merged IoT courses on their academic programs (i.e., biased coverage mode) or developed a complete degree program (i.e., full coverage). In the first case, the universities focus on specific IoT technologies exploiting knowledge from broader disciplines such as Computer Science (CS), Electronics Engineering (EE), Communication Engineering, and Automation Engineering. These courses emphasize IoT sensing and control technologies, like sensors, RFID, microcomputers, embedded systems, IoT networking, IoT wireless communications, information sensing, and processing. Regarding the second group of universities, fundamental courses inherited either from CS or EE or both are taught as well as new courses such as “Introduction to IoT”, “IoT sensing technology”, “IoT control system and technology” and “IoT systems integration and application”.

Regarding the fourth research question (**RQ4**), EC is weakly presented in teaching programs since 22% of the reviewed university courses contain topics devoted to EC (see Figure 4) while only 9% of the interviewed academics teach EC (see Figure 6). Precisely, academics teach to a great or to a very great extent EC general concept, EC applications as well as EC speed and efficiency (see Table 22). Technologies used in EC computing implementations such as Mobile Edge Computing, Fog computing, Service composition and service-oriented computing, Micro data centers, and Container technology are taught equally. At the same time, the Azure edge is discussed the least concerning other technologies (Table 23). We also did not find related works in the literature that deal with the inclusion of EC-related topics in higher education curricula. Consequently, researchers need to identify the industry needs for EC knowledge and develop related study courses to introduce relevant topics into the curricula.

Regarding the fifth research question (**RQ5**), the primary teaching method is lectures, then project-based learning with teamwork. The least used technique is problem-based learning since teachers have a low understanding of real industrial problems that could be analyzed with students. The mentioned conclusions result from data presented in Table 3, Table 16, and Table 20.

Regarding the sixth research question (**RQ6**), according to the researchers’ responses, the educational process does not focus on teaching how industrial problems can be solved with AI, IoT, or EC. Specifically, the majority of educators do not focus on teaching AI techniques for applications in deliveries, cognitive systems, supply chain management, and quality problems. Respectively, as far as IoT is concerned, a high percentage of academics have not included the teaching of IoT application in market behavior, deliveries, robotics, quality problems, and machine condition monitoring in their courses. Given the EC, the number of academics teaching it is not adequate. Besides, it is also worth noting that there is not a sufficient number of practical courses where students can apply these technologies properly in universities to prepare for the labor market. Therefore, the universities and institutions have to bridge the gap between theory and practice, as well as develop a modern curriculum with relevant courses that can meet the real market and industry needs.

However, apart from the results of this study, similar conclusions have emerged from other relevant research. For example, the report [63] of the European Commission on skills for Industry 4.0 argues that the current educational programs insufficiently address critical areas such as modeling and simulation of manufacturing processes, equipment running, troubleshooting, integration skills, quality management, emergency management, industrial hygiene, risk assessment. Additionally, most academics do not have prior experience in the industry, thus not delivering experience-led teaching [64]. However, a significant barrier to implementing new teaching methodologies is the lack of adequate capital and regular funding of educational institutes.

The universities and institutions have to build connections between theory and practice and develop a modern curriculum with the most relevant courses that meet the real needs of the market and industry. At the same time, in the context of the educational process’ alignment with the industrial needs, academics should collaborate with engineers from industry on project-based learning approaches while bringing industrial practitioners into universities to conduct lectures that will improve students’ practical skills. Nevertheless, to achieve all of the above, there must be adequate financial support from government agencies in educational institutions.

Regarding the last research question (**RQ7**), teachers need support with examples of the practical application of AI, IoT, and EC, and they need in particular:

1. Examples of computer vision, natural language processing and cognitive computing applications in the industry.
2. Examples of applications of generative adversarial networks and transformer to solve practical problems from industry.
3. Bound data mining to industry examples to show the importance of business understanding and deployment phases.
4. Examples of applications of fuzzy systems and genetic algorithms in computational intelligence.
5. Examples of natural language processing application.
6. Examples of applications of computer vision and cognitive computing in Industry 4.0.
7. Examples of applications AI in cognitive systems, supply chains management, delivery in Industry 4.0.
8. Examples of AI applications in Industry 4.0 which can be used for workshops and problem - based learning.
9. Examples of security issues related to Internet of Things.
10. Examples of issues concerning market behavior and deliveries in the IoT context.
11. Examples of current deployment of IoT products and technologies in the production environment.
12. Examples of IoT applications in Industry 4.0 which can be used for workshops and problem - based learning.
13. Examples of processing data gathered by intelligent sensors in IoT area.
14. Examples of edge computing possible applications in industry.

Industrial examples of AI, IoT, and EC can also support academics in better understanding of the topics to be better prepared for the teaching process. It should be emphasized that the discussed technologies are new, and in many cases, teachers must learn them by themselves before they start teaching students. Therefore, academics need to collaborate with industry so that teachers understand what the new technologies can be used for. Besides, teaching by example always produces the best results. Additionally, teachers need comprehensive support in the literature describing applications examples of the discussed technologies in solving practical problems.

## 7. Recommendations

The proposed research methodology has provided a global vision of the IoT, AI and EC knowledge and skills being taught in academic institutions around Europe (and not only), which was the study’s object. The collection and analysis of the data from the surveys have given us answers to the research questions discussed in the previous section. These findings highlight the need for certain aspects of the study to be addressed by academics to offer better training for future Industry 4.0 workers. This section discusses potential solutions and strategies to respond to our study’s needs, obstacles, and opportunities.

As shown in the previous sections, most of the existing study programs do not currently cover the pillars of Industry 4.0 (Artificial Intelligence, Internet of Things, and Edge Computing) evenly. In particular, the IoT and EC are considerably studied less than AI. Not surprisingly, this finding—which was already identified in the surveys conducted—also appears when analyzing the interest over time of these three topics in Google Trends.

**Figure 9:**
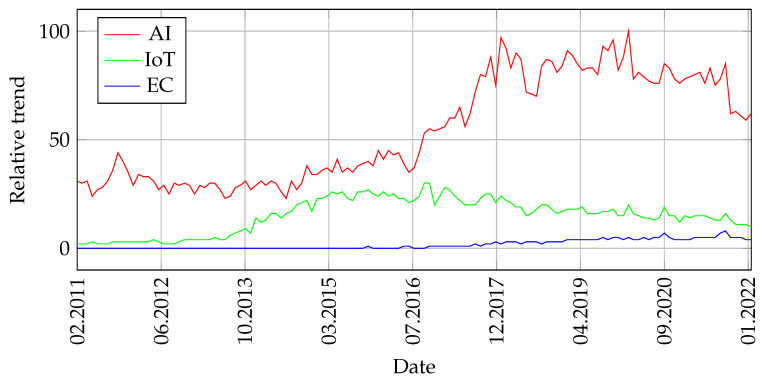
Interest over time in the terms “Internet of Things”, “Artificial Intelligence”, and “Edge Computing” according to Google Trends. Data Source: Google Trends (https://trends.google.com/trends), access date: 1 February 2022.

As shown in Figure 9, since 2011, the number of Google queries about “AI” has been considerably higher than the number of queries involving “IoT” and/or “EC”. This is most probably related to the maturity and real-world impact of these terms: while AI has been used since the 1960s [65], the terms Internet of Things and Edge Computing were coined almost 40 years later [20,66]. It is safe to say that nowadays, EC and IoT are still evolving and finding their place in the industrial market.

This “ever-evolving” trend hinders the incorporation of these topics (i.e., IoT and EC) in academic curricula as what may happen is that by the time new curricula have been updated and approved, the recently added topics may be outdated. In other words, in the time required to prepare new content, train faculty members and obtain approval and/or accreditation, the newly incorporated content is no longer applicable or useful for students. Therefore, several techniques related to IoT and EC methods or areas (such as cybersecurity in the context of IT, market and delivery behavior in the IoT, current IoT products and technologies, or an analysis of IoT sensors’ data processing) are not adequately covered in the contents. Neglecting these crucial topics in the contents of the subjects implies the omission of exercises, case studies, practical cases, etc. (such as references to computer vision, natural language processing, cognitive computing, generative adversarial networks, and IoT applications), which would contribute to effectively training students to address the challenges posed by Industry 4.0 successfully.

To cover this need, the authors of this paper propose that the continuous improvement processes—that are in charge of reviewing and updating contents in higher education study programs—focus more specifically on: (1) empowering collaboration models with external agents, (2) adapting the technological infrastructure, and (3) training of the teaching faculty. These three strategies are interrelated: while the first two may directly influence each other’s evolution, they could also influence the third one.

To properly develop collaboration with external agents, especially with former students [67], employers and stakeholders from different industry sectors [68,69,70] are considered vital. This strategy creates effective opportunities to design curriculum, identify strengths/weaknesses, determine to what extent skills being taught meet real-world needs, discover current/future trends, and even innovate and develop new teaching dynamics. Furthermore, apart from contributing to the design and definition/refinement of contents [67], such strategies can derive into workshops, class projects [71], case studies, challenges such as a Final Thesis with expert mentoring [72], internship opportunities, etc. This clearly shows how concepts can be incorporated into real-world scenarios and how representatives from the fourth industrial revolution can be involved in the training of future workers. In [71], the project-based industries are presented as dynamics that provide specific benefits in terms of knowledge and understanding business needs. They enable knowledge and skills to be applied in a professional environment. Knowledge and technology transfer are encouraged simultaneously, thus promoting industrial innovation. In [69], the benefits and motivations for both parties involved, the University and industry, are described in financial, technological, and strategic terms:

- For universities, the availability of new funding, the use of new infrastructures or materials, the possibility of incorporating professional experience and new options to offer a more practical learning methodology.
- For companies, cost reduction, risk sharing, access to new resources, headhunting, bringing naive, fresh views (i.e., from students) to existing projects, and gaining a more accurate vision of technological trends.

Universities have to consider that the benefits brought about by such collaborations could lead to changes in the official definition of study programs and the planning and description of teaching duties. The official definition could be subject to modification due to the remodeling of certain aspects of the program, such as specific competencies, modules, course and subject content, content describers, marking criteria and assessment guides, training activities, and/or teaching methodologies. In terms of responsibilities, obstacles to a new form of cooperation would undoubtedly arise, requiring time and effort to be overcome. For example, the industry may favor developing work in a specific area. At the same time, the universities may opt for another area, the industry may restrict access to specific information that teaching faculty may deem necessary for the creation of an effective learning methodology, or there could be discrepancies in the time scales envisioned by one of the two parties [69]. These factors motivate the creation of long-term collaboration agreements between industry and universities—without forgetting the necessary confidentiality clauses and non-disclosure agreements to protect companies’ Intellectual Property.

The intrinsic characteristics (e.g., lack of standardization, heterogeneity, maintainability) of the required infrastructure for teaching the concepts associated with Industry 4.0 (IoT, AI, and EC) may initially be barriers for universities. While acquiring AI knowledge and its practical application may seem feasible through well-known software libraries (e.g., PyTorch, TensorFlow, sci-kit-learn, weka) and general-purpose computers, the IoT requires certain elements and technologies which are not readily available. In the case of IoT, the key elements [73] to develop knowledge and skills are: Identification, Sensing, Communication, Computation, Services, and Semantics, which require technology such as sensors, wearables, actuators, Arduino, Raspberry Pi, or Intel Galil. In light of this, universities must invest in the specific design of their laboratories [74,75]. For example, in [74], various phases of the creation of an introductory IoT laboratory are exemplified, while [75] shows the results of a project based on the design of IoT exercises which incorporate the use of a wide range of devices (such as Arduino, Voting machine, plane spotter, temperature monitor, object tracking). In [73], an IoT laboratory is created (with Arduino, sensors, transducers, actuators) to enable the practical teaching of several concepts of different subjects (such as computer architecture and distributed systems). Similarly, in [76], the use of Virtual Reality technology is presented in an application for mobile devices to facilitate a better and more adequate understanding of the functionalities of Raspberry Pi. In order to obtain the financial resources needed to invest in such equipment, the universities should consider the advantages that working with industrial companies, institutions, and government administration can bring in terms of access to resources/infrastructure, grants, and new sources of funding. Notwithstanding, IoT and EC pose several challenges when conceiving a hands-on learning environment for practical training in these areas. First and foremost, these are transversal topics that cover different knowledge areas traditionally set apart in engineering curricula [77]. This approach limits the capability of classic academic programs to deliver in-depth knowledge in this area. For instance, IoT covers concepts ranging from computer engineering to telecommunications engineering, including electronics and cybersecurity. Similarly, EC covers concepts ranging from computer science to telecommunications engineering, including DevOps or computer engineering. These concepts are difficult to find in undergraduate programs where students focus on a single discipline (e.g., computer engineering). Second, despite the latest efforts to use Virtual Reality in IoT training, the conception of a virtual lab in EC and/or IoT is still far from reality. Especially compared to AI, it is possible to set up a powerful and low-cost training environment in less than five minutes [78] with little (or no) technical knowledge regarding hardware configuration or software package installation. Deploying and maintaining an IoT and/or EC laboratory would require a wide span of knowledge that might be difficult to find. Last but not least, as far as budgets are concerned, IoT and EC devices are exposed to real-world inclemency (e.g., students that apply wrong voltages). Although this also happens in any other type of laboratory. Fixing the catastrophes of the real-world inclemency in an IoT laboratory is considerably harder for students than rebooting a computer, deploying a disk image on a hard drive, or even replacing a general-purpose computer (IoT devices, such as Raspberry Pi, may take months to be delivered). Indeed, academic programs cannot afford laboratory downtimes. This situation motivates the need of conceiving a (virtual) IoT and/or EC laboratory in which interdisciplinary students can learn, experiment, and innovate safely and effectively, fostering cross-layer interactions between students that would make them better suited to address present and future Industry 4.0 real-world demands.

Proper training of the teaching faculty is of paramount importance when responding to these needs. Teacher training goes hand-in-hand with the incorporation of new content and vice-versa. Better prepared teachers could, in turn, create and develop more innovative content. Once again, the challenge that needs to be addressed is the ever-evolving nature of the concepts associated with AI, IoT, and EC. That is, during the time that it takes to prepare new content for teacher training and train the teaching staff, these contents might become outdated. Therefore, it is necessary to develop an agile methodology that can keep up with the pace of this evolution and effectively deliver the content to students. In this regard, we envisage the need for a strong collaboration between industry partners—who are aware of market needs—research scientists—who are aware of the latest technology achievements—and academics—who are aware of the most effective pathways to convey knowledge to students.

Notwithstanding, it is worth considering that the recommendations discussed in this section might not be applicable for all universities worldwide. Industry 4.0 training requires a unique ecosystem in which companies (offering novel problems and challenges), academic institutions (continuously adapting their curricula to meet market demands), and students (willing to solve real-world problems) actively interact. For those environments in which this ecosystem is unfeasible, this section provides a modest pathway toward the definition of future improvement actions. In addition, the digitization of modern education may contribute to fighting this situation. The authors, within the framework of the Erasmus+ PLANET4 project, propose a blended (i.e., online + face-to-face) training course that integrates AI in Industry 4.0, with a focus on the AC, EC, and IoT technologies, including: (1) e-learning courses on the theory of AI and ML on the Edge Technologies, (2) hands-on workshop for solving real industrial challenges, and (3) Innovation and Change Management in Industry 4.0 training workshops. The training aspects are described in more detail in Section 8.2.

## 8. Conclusions

This sections discusses the limitations of the presented research and proposes some future work directions to extend this work.

### 8.1. Work Limitations

The first limitation identified in this work can be derived from Figure 2 and Figure 5. That is, collected data cover selected countries from Europe. This is because we have found that it is unfeasible to reach the personnel associated with AI, IoT, and EC teaching areas from all the universities reliably and efficiently. We have seen that several universities do not publicly share the instructors’ (or Program Coordinators) names/email addresses on their course programs’ websites, or the available data placed there are wrong/misleading. Maybe, this could be motivated by the constant role changes in terms of training activities to which academic staff is exposed. Also, the number of interviewed academics from the same university is relatively small, which might give partial or biased views of the reality. This is because the knowledge areas (i.e., AI, IoT, EC) are very specific and, thus, there is typically a small amount of personnel associated with them. However, we believe that the amount of data that we have collected for this work can capture the current status of Higher Education regarding these scientific fields. At the same time, one can see that the geographical distribution of academics is not uniform. Knowing that the quality of education differs significantly from country to country, it can quickly be concluded that the research results are not trustworthy. However, analyzing them, we have seen similar trends between data from different countries, which endorses the reliability of this study.

Similarly, we have examined training programs from selected universities in Europe. Again, up-to-date syllabus details regarding these programs are often unavailable on public websites. We understand that some universities see their syllabus as their Intellectual Property and, thus, are averse to sharing this information in an out-of-control way, fearing other universities to *copy* their training programs. However, we believe that a training program is far more than a course syllabus and involves teaching methodologies, instructor skill set, available facilities (e.g., laboratories), university added-value services, etc. Therefore, we think that making the information related to training programs publicly available would improve similar training programs from different universities (i.e., competency) and would ease the exchange of students among universities.

This leads to the following limitation of this work. As envisaged in Figure 9, Artificial Intelligence, Internet of Things, and Edge Computing are fields under constant evolution. Day to day, new algorithms, technologies, and use-cases emerge as promising alternatives to the current developments achieved so far. Typically, these advancements shape the evolution of training programs. Therefore, the results presented in this work are limited to showing the current picture of training in AI, IoT, and EC nowadays. Nonetheless, these results should be taken as a starting point to build training programs for shaping the future professionals of Industry 4.0.

### 8.2. Future Research

In order to address the limitations identified in the previous section, we propose a two-fold approach. On the one hand, the number of universities involved in this research could be increased by taking advantage of already existing communication channels, such as the Erasmus+ framework, which connects training centers all over Europe. On the other hand, setting up a virtual environment in which educational institutions would be encouraged to upload the details—maybe in standardized public repositories—of their training programs would ease the task of mapping academics and their skills.

In this work, we have explored the current role of academics in transferring knowledge and skills on Artificial Intelligence, Internet of Things, and Edge Computing for Industry 4.0. In terms of hard skills, we have identified that IoT and EC are covered with less emphasis than AI (see Figure 4 and Figure 6). In terms of soft skills, we have seen that the field of Innovation and Change Management (I&CM) (i.e., properly managing the adoption of innovative solutions to reshape industrial processes) is not broadly incorporated. This conclusion comes from the observation that topics related to AI, IoT, and EC are not related to I&CM topics in university training programs. Therefore, it might be interesting to develop a multidisciplinary training program in which students (from both academia and industry) gain the necessary skills to bring value to Industry 4.0. Specifically, this course could start with an Innovation and Change Management workshop in which groups of students would (1) propose innovative ideas to solve Industry 4.0 challenges, (2) learn to distinguish what is innovative, and (3) come up with the best way to implement—from a perspective of both management and technical—these innovations in the industrial process. Next, students would learn all the necessary skills to bring the latest achievements in AI, IoT, and EC to real-world Industry 4.0 use-cases. This part should be split into two sections: the first one aimed to train the technology fundamentals, and the second part aimed to develop hands-on experience with these technologies. Finally, the course could end up with a Final Project mentored by an industry expert that would bring her/his insights and experience to the student.

To sum up, this work considers the challenge of Industry 4.0 training from the academics (partial) perspective. Conducting similar research from the industrial and students’ points of view could contribute to obtaining (and complementary) a holistic view of the whole situation. This would enable practitioners to draw broad conclusions that would encompass the main stakeholders of training in Industry 4.0.

## Figures and Tables

**Table 1:** Programming languages used in AI, IoT, and EC courses.

Programming Language	AI Courses		IoT Courses		EC Courses	
	Number	Percentage	Number	Percentage	Number	Percentage
C or C++	1	2%	1	7%	0	0%
Python	7	13%	4	27%	4	36%
Java	1	2%	1	7%	1	9%
Matlab	8	15%	1	7%	0	0%
Prolog	1	2%	0	0%	0	0%
R	6	11%	1	7%	1	9%
SQL	3	6%	0	0%	0	0%
No data	27	50%	7	47%	5	45%

**Table 2:** Software and environments used in AI, IoT, and EC courses.

Software/ Environment	AI Courses		IoT Courses		EC Courses	
	Number	Percentage	Number	Percentage	Number	Percentage
Aitech Sphinx	5	8%	0	0%	0	0%
Statistica	2	3%	0	0%	0	0%
Matlab	8	14%	1	8%	0	0%
Fuzzy logic toolbox	3	5%	0	0%	0	0%
Tableu	1	2%	1	8%	1	8%
Azure services	0	0%	2	15%	2	17%
Prolog	1	2%	0	0%	0	0%
PyTorch	1	2%	0	0%	1	8%
Arduino SDK	0	0%	2	15%	1	8%
Octave	3	5%	0	0%	0	0%
Tensorflow	2	3%	0	0%	1	8%
Docker	0	0%	0	0%	1	8%
IBM SPSS	1	2%	0	0%	0	0%
No data	32	54%	7	54%	5	42%

**Table 3:** Teaching methodologies of AI, IoT, and EC courses.

Software/ Environment	AI Courses		IoT Courses		EC Courses	
	Number	Percentage	Number	Percentage	Number	Percentage
Lectures	39	46%	35	41%	36	42%
Lab activity	28	33%	27	31%	27	32%
Problem-based learning	1	1%	0	0%	0	0%
Project-based learning	11	13%	18	21%	16	19%
No data	6	7%	6	7%	6	7%

**Table 4:** Disciplines of AI in teaching, 100% = 69.

To What Extent Do You Teach the Following Disciplines in the Educational Process of AI?	Not at All	To a Small Extent	To Some Extent	To a Moderate Extent	To a Great Extent	To a Very Great Extent	No Answer
Philosophy of mind	**44.9%**	30.4%	13.0%	5.8%	1.4%	0.0%	4.3%
Cognitive modeling	**31.9%**	**31.9%**	20.3%	2.9%	7.2%	1.4%	4.3%
Mathematics	14.5%	14.5%	**26.1%**	21.7%	10.1%	11.6%	1.4%
Logic	8.7%	**31.9%**	20.3%	15.9%	10.1%	8.7%	4.3%
Linguistics	**37.7%**	30.4%	8.7%	11.6%	2.9%	1.4%	7.2%
Computer science	4.3%	8.7%	7.2%	10.1%	**34.8%**	**34.8%**	0.0%

**Table 5:** Areas of AI in teaching, 100% = 69.

To What Extent Do You Teach the Following AI Areas?	Not at All	To a Small Extent	To Some Extent	To a Moderate Extent	To a Great Extent	To a Very Great Extent	No Answer
Machine Learning	2.9%	10.1%	15.9%	20.3%	**26.1%**	23.2%	1.4%
Deep Learning	**21.7%**	18.8%	8.7%	17.4%	17.4%	14.5%	1.4%
Data mining	11.6%	**20.3%**	18.8%	15.9%	**20.3%**	8.7%	4.3%
Computational intelligence	13.0%	17.4%	15.9%	**20.3%**	15.9%	11.6%	5.8%
Natural language processing	**43.5%**	18.8%	14.5%	11.6%	4.3%	2.9%	4.3%
Computer vision	**39.1%**	13.0%	15.9%	10.1%	11.6%	8.7%	1.4%
Cognitive computing	**42.0%**	24.6%	17.4%	8.7%	1.4%	1.4%	4.3%

**Table 6:** Basic Machine Learning techniques in teaching, 100% = 69.

To What Extent Do You Teach the Basic Machine Learning Techniques?	Not at All	To a Small Extent	To Some Extent	To a Moderate Extent	To a Great Extent	To a Very Great Extent	No Answer
Supervised learning	8.7%	8.7%	8.7%	20.3%	21.7%	**31.9%**	0.0%
Semi-supervised learning	**27.5%**	21.7%	11.6%	17.4%	10.1%	8.7%	2.9%
Unsupervised learning	8.7%	21.7%	10.1%	17.4%	**23.2%**	17.4%	1.4%
Reinforcement learning	26.1%	**30.4%**	10.1%	17.4%	7.2%	5.8%	2.9%

**Table 7:** Major Deep Learning models in teaching, 100% = 69.

To What Extent Do You Teach the Major Deep Learning Models?	Not at All	To a Small Extent	To Some Extent	To a Moderate Extent	To a Great Extent	To a Very Great Extent	No Answer
Convolution neural network	**23.2%**	17.4%	7.2%	18.8%	18.8%	13.0%	1.4%
Recurrent neural network	**24.6%**	23.2%	8.7%	21.7%	11.6%	10.1%	0.0%
Transformer	**47.8%**	18.8%	14.5%	11.6%	4.3%	0.0%	2.9%
Generative adversarial network (GAN)	**50.7%**	17.4%	10.1%	8.7%	7.2%	1.4%	4.3%

**Table 8:** Data mining phases, 100% = 69.

To What Extent Do You Teach the Following Data Mining Phases (CRISP-DM)?	Not at All	To a Small Extent	To Some Extent	To a Moderate Extent	To a Great Extent	To a Very Great Extent	No Answer
Business understanding	**42.0%**	20.3%	7.2%	14.5%	13.0%	0.0%	2.9%
Data understanding	14.5%	13.0%	15.9%	**20.3%**	23.2%	8.7%	4.3%
Data preparation	14.5%	11.6%	**23.2%**	14.5%	20.3%	13.0%	2.9%
Modeling	5.8%	10.1%	20.3%	**24.6%**	18.8%	17.4%	2.9%
Evaluation	13.0%	8.7%	17.4%	**21.7%**	20.3%	14.5%	4.3%
Deployment	21.7%	**23.2%**	17.4%	14.5%	15.9%	1.4%	5.8%

**Table 9:** Computational intelligence issues, 100% = 69.

To What Extent Do You Teach Computational Intelligence Issues?	Not at All	To a Small Extent	To Some Extent	To a Moderate Extent	To a Great Extent	To a Very Great Extent	No Answer
Fuzzy systems	**39.1%**	21.7%	10.1%	11.6%	7.2%	5.8%	4.3%
Neural networks	7.2%	13.0%	14.5%	18.8%	23.2%	**23.2%**	0.0%
Genetic algorithms	**30.4%**	20.3%	11.6%	13.0%	8.7%	10.1%	5.8%

**Table 10:** Natural language processing issues, 100% = 69.

To What Extent Do You Teach Natural Language Processing Issues?	Not at All	To a Small Extent	To Some Extent	To a Moderate Extent	To a Great Extent	To a Very Great Extent	No Answer
Speech recognition	**62.3%**	11.6%	10.1%	8.7%	1.4%	2.9%	2.9%
Natural language generation	**66.7%**	13.0%	7.2%	4.3%	4.3%	1.4%	2.9%
Natural language translation	**68.1%**	14.5%	7.2%	2.9%	2.9%	1.4%	2.9%

**Table 11:** Computer vision issues, 100% = 69.

To What Extent Do You Teach Computer Vision Issues?	Not at All	To a Small Extent	To Some Extent	To a Moderate Extent	To a Great Extent	To a Very Great Extent	No Answer
Image classification	**34.8%**	11.6%	14.5%	10.1%	13.0%	15.9%	0.0%
Object localization and detection	**40.6%**	17.4%	5.8%	15.9%	11.6%	5.8%	2.9%
Image segmentation	**44.9%**	11.6%	13.0%	5.8%	13.0%	10.1%	1.4%
Domain adaptation	**60.9%**	14.5%	7.2%	8.7%	1.4%	4.3%	2.9%
Neural style transfer	**63.8%**	14.5%	7.2%	7.2%	4.3%	1.4%	1.4%

**Table 12:** Cognitive computing issues, 100% = 69.

To What Extent Do You Teach Cognitive Computing Issues?	Not at All	To a Small Extent	To Some Extent	To a Moderate Extent	To a Great Extent	To a Very Great Extent	No Answer
Interactive task learning	**52.2%**	21.7%	7.2%	5.8%	8.7%	0.0%	4.3%
Game playing agents	**58.0%**	15.9%	11.6%	4.3%	8.7%	0.0%	1.4%
Meta-algorithms in cognitive computing	**58.0%**	29.0%	5.8%	1.4%	2.9%	0.0%	2.9%

**Table 13:** Programming languages for AI applications, 100% = 69.

To What Extent Do You Teach the Following Programming Languages for AI Applications?	Not at All	To a Small Extent	To Some Extent	To a Moderate Extent	To a Great Extent	To a Very Great Extent	No Answer
C or C++	**50.7%**	8.7%	8.7%	10.1%	8.7%	7.2%	5.8%
Python	8.7%	**23.2%**	13.0%	14.5%	15.9%	**23.2%**	1.4%
Lisp	**73.9%**	7.2%	2.9%	1.4%	4.3%	0.0%	10.1%
Java	**49.3%**	11.6%	8.7%	11.6%	4.3%	4.3%	10.1%
Matlab	**37.7%**	10.1%	5.8%	14.5%	8.7%	15.9%	7.2%
Prolog	**63.8%**	4.3%	2.9%	5.8%	7.2%	5.8%	10.1%
R	**63.8%**	13.0%	4.3%	4.3%	1.4%	1.4%	11.6%

**Table 14:** Software/environment in AI teaching, 100% = 69.

To What Extent Do You Use the Following Software/Environment to Teach AI?	Not at All	To a Small Extent	To Some Extent	To a Moderate Extent	To a Great Extent	To a Very Great Extent	No Answer
AITECH SPHINX	**81.2%**	2.9%	0.0%	1.4%	1.4%	0.0%	13.0%
Statistica	**71.0%**	4.3%	1.4%	2.9%	4.3%	1.4%	14.5%
Matlab	**34.8%**	11.6%	8.7%	10.1%	11.6%	17.4%	5.8%
MS Excel	**36.2%**	21.7%	10.1%	8.7%	8.7%	2.9%	11.6%
Scilab	**75.4%**	7.2%	1.4%	2.9%	0.0%	1.4%	11.6%
RStudio	**66.7%**	8.7%	1.4%	2.9%	2.9%	1.4%	15.9%
SWI Prolog	**63.8%**	10.1%	2.9%	1.4%	7.2%	4.3%	10.1%
PyCharm	**58.0%**	13.0%	5.8%	5.8%	4.3%	4.3%	8.7%
Spyder	**58.0%**	10.1%	2.9%	1.4%	10.1%	2.9%	14.5%
Visual Studio Code	**52.2%**	8.7%	5.8%	11.6%	5.8%	2.9%	13.0%

**Table 15:** Teaching the applications of AI, 100% = 69.

To What Extent Do You Teach AI in the Following Applications?	Not at All	To a Small Extent	To Some Extent	To a Moderate Extent	To a Great Extent	To a Very Great Extent	No Answer
Manufacturing processes monitoring	**44.9%**	13.0%	13.0%	10.1%	5.8%	7.2%	5.8%
Deliveries	**65.2%**	4.3%	13.0%	1.4%	2.9%	4.3%	8.7%
Computer vision	**44.9%**	13.0%	13.0%	10.1%	5.8%	7.2%	5.8%
Scheduling problems	**65.2%**	4.3%	13.0%	1.4%	2.9%	4.3%	8.7%
Predictive maintenance	**42.0%**	7.2%	15.9%	4.3%	10.1%	14.5%	5.8%
Quality problems	**40.6%**	13.0%	14.5%	14.5%	5.8%	1.4%	10.1%
Supply chains management	**42.0%**	15.9%	13.0%	11.6%	4.3%	5.8%	7.2%
Anomaly detection	**46.4%**	11.6%	11.6%	17.4%	1.4%	2.9%	8.7%
Optimization	**47.8%**	18.8%	7.2%	7.2%	4.3%	1.4%	13.0%
Cognitive systems	**31.9%**	11.6%	18.8%	10.1%	15.9%	2.9%	8.7%
Autonomous systems	**34.8%**	23.2%	8.7%	5.8%	13.0%	8.7%	5.8%
Robots	**40.6%**	13.0%	11.6%	7.2%	10.1%	11.6%	5.8%

**Table 16:** Learning techniques in AI teaching, 100% = 69.

To What Extent Do You Use the Following Learning Techniques to Teach AI?	Not at All	To a Small Extent	To Some Extent	To a Moderate Extent	To a Great Extent	To a Very Great Extent	No Answer
Lectures	0.0%	5.8%	10.1%	15.9%	**36.2%**	31.9%	0.0%
Labs	8.7%	4.3%	17.4%	14.5%	**31.9%**	20.3%	2.9%
Workshops	0.0%	5.8%	10.1%	15.9%	**36.2%**	31.9%	0.0%
Problem based learning	18.8%	2.9%	15.9%	13.0%	**24.6%**	20.3%	4.3%
E-learning	18.8%	8.7%	10.1%	14.5%	**21.7%**	**21.7%**	4.3%
Project Based Learning (team work)	**33.3%**	10.1%	11.6%	14.5%	11.6%	10.1%	8.7%
Project Based Learning (individual work)	8.7%	4.3%	17.4%	14.5%	**31.9%**	20.3%	2.9%

**Table 17:** Teaching topics in the area of IoT, 100% = 31.

To What Extent Do You Teach the Following Topics in the Area of IoT?	Not at All	To a Small Extent	To Some Extent	To a Moderate Extent	To a Great Extent	To a Very Great Extent	No Answer
General introduction to IoT	6.5%	3.2%	16.1%	**32.3%**	22.6%	16.1%	3.2%
Application scenarios of IoT	9.7%	9.7%	16.1%	16.1%	**25.8%**	16.1%	6.5%
IoT architecture	12.9%	9.7%	22.6%	19.4%	**25.8%**	6.5%	3.2%
IoT deployment	**22.6%**	16.1%	16.1%	12.9%	**19.4%**	6.5%	6.5%
IoT components	16.1%	16.1%	**19.4%**	12.9%	**19.4%**	12.9%	3.2%
M2M industrial IoT protocols	**35.5%**	16.1%	3.2%	19.4%	16.1%	6.5%	3.2%
Sensors	6.5%	**22.6%**	9.7%	**22.6%**	12.9%	16.1%	9.7%
Application development	16.1%	**22.6%**	12.9%	6.5%	19.4%	16.1%	6.5%
IoT maintenance	**29.0%**	32.3%	16.1%	3.2%	9.7%	3.2%	6.5%
Computer Networking	**38.7%**	19.4%	9.7%	9.7%	9.7%	9.7%	3.2%
Distributed processing in IoT networks	**35.5%**	9.7%	22.6%	19.4%	6.5%	3.2%	3.2%
Data analytics	**25.8%**	9.7%	9.7%	25.8%	12.9%	9.7%	6.5%
Cloud computing	**25.8%**	16.1%	19.4%	6.5%	16.1%	12.9%	3.2%
Databases development	22.6%	**25.8%**	9.7%	16.1%	9.7%	9.7%	6.5%
Data transfer protocols	**29.0%**	16.1%	12.9%	16.1%	16.1%	0.0%	9.7%
Knowledge management	**32.3%**	9.7%	12.9%	12.9%	22.6%	3.2%	6.5%
Cybersecurity	**35.5%**	22.6%	9.7%	12.9%	12.9%	3.2%	3.2%
Cryptography	**54.8%**	16.1%	3.2%	12.9%	3.2%	0.0%	9.7%
Basic network attacks	**48.4%**	22.6%	3.2%	9.7%	3.2%	6.5%	6.5%
Real time operating systems	**38.7%**	19.4%	9.7%	16.1%	9.7%	0.0%	6.5%
Searching for vulnerabilities	**58.1%**	12.9%	0.0%	12.9%	9.7%	0.0%	6.5%
Computer Networking	**41.9%**	16.1%	3.2%	16.1%	9.7%	9.7%	3.2%
IoT communication terminals and gateways	**29.0%**	16.1%	16.1%	19.4%	12.9%	0.0%	6.5%

**Table 18:** Context of Teaching the IoT, 100% = 31.

To What Extent Do You Teach IoT in the Following Context?	Not at All	To a Small Extent	To Some Extent	To a Moderate Extent	To a Great Extent	To a Very Great Extent	No Answer
Quality problems	**38.7%**	12.9%	22.6%	3.2%	9.7%	6.5%	6.5%
Machine condition monitoring	**32.3%**	22.6%	3.2%	12.9%	16.1%	6.5%	6.5%
Robotics	**41.9%**	9.7%	6.5%	12.9%	12.9%	12.9%	3.2%
Deliveries	**48.4%**	12.9%	12.9%	6.5%	6.5%	6.5%	6.5%
Market behavior	**58.1%**	12.9%	3.2%	9.7%	6.5%	3.2%	6.5%
Data management	19.4%	12.9%	16.1%	**29.0%**	12.9%	3.2%	6.5%
Support decision-making	**25.8%**	12.9%	12.9%	19.4%	22.6%	3.2%	3.2%
Process parameters monitoring	**29.0%**	12.9%	3.2%	12.9%	22.6%	9.7%	9.7%
Logistics	**29.0%**	19.4%	16.1%	9.7%	12.9%	6.5%	6.5%

**Table 19:** Teaching applications of IoT in different technologies, 100% = 31.

To What Extent Do You Teach IoT in the Following Techniques?	Not at All	To a Small Extent	To Some Extent	To a Moderate Extent	To a Great Extent	To a Very Great Extent	No Answer
Data processing and transformation	9.7%	16.1%	**22.6%**	**22.6%**	16.1%	6.5%	6.5%
Data display	**25.8%**	9.7%	22.6%	12.9%	12.9%	9.7%	6.5%
Industrial automation	**32.3%**	9.7%	25.8%	6.5%	9.7%	9.7%	6.5%
Anomaly detection	**38.7%**	16.1%	6.5%	12.9%	16.1%	3.2%	6.5%
IaaS (Infrastructure as a Service)	**51.6%**	16.1%	6.5%	6.5%	6.5%	9.7%	3.2%
PaaS (Platform as a Service	**41.9%**	19.4%	12.9%	6.5%	12.9%	3.2%	3.2%
SaaS (Software as a Service)	**45.2%**	16.1%	9.7%	6.5%	16.1%	3.2%	3.2%
Application Programming Interface	**32.3%**	9.7%	16.1%	16.1%	9.7%	9.7%	6.5%
Digital twins	**32.3%**	19.4%	12.9%	12.9%	9.7%	6.5%	6.5%
Big data management	**32.3%**	19.4%	22.6%	6.5%	9.7%	3.2%	6.5%
Containers and orchestrators (Docker, Swarm, Kubernetes, EKS…)	**54.8%**	9.7%	9.7%	9.7%	6.5%	3.2%	6.5%

**Table 20:** Learning techniques in IoT Teaching, 100% = 31.

To What Extent Do You Use the Following Learning Tools to Teach IoT?	Not at All	To a Small Extent	To Some Extent	To a Moderate Extent	To a Great Extent	To a Very Great Extent	No Answer
Lectures	0.0%	9.7%	12.9%	19.4%	25.8%	**32.3%**	0.0%
Labs	25.8%	3.2%	9.7%	9.7%	**29.0%**	19.4%	3.2%
Workshops	**29.0%**	6.5%	19.4%	16.1%	6.5%	12.9%	9.7%
Problem Based Learning	**35.5%**	9.7%	12.9%	3.2%	19.4%	12.9%	6.5%
E-learning	**29.0%**	6.5%	16.1%	16.1%	6.5%	16.1%	9.7%
Project Based Learning (team work)	22.6%	9.7%	9.7%	9.7%	19.4%	**25.8%**	3.2%
Project Based Learning (individual work)	16.1%	6.5%	**22.6%**	19.4%	12.9%	19.4%	3.2%

**Table 21:** Software/technology in IoT teaching, 100% = 31.

To What Extent Do You Use the Following Software/Technology to Teach IoT?	Not at All	To a Small Extent	To Some Extent	To a Moderate Extent	To a Great Extent	To a Very Great Extent	No Answer
MapReduce	**67.7%**	12.9%	6.5%	3.2%	3.2%	0.0%	6.5%
AWS Lambda	**77.4%**	6.5%	0.0%	6.5%	0.0%	3.2%	6.5%
Azure functions	**61.3%**	16.1%	9.7%	0.0%	3.2%	3.2%	6.5%
Arduino IoT	**38.7%**	6.5%	16.1%	0.0%	16.1%	19.4%	3.2%
Cloud Services & Serverless Technologies (AWS, GCP, DigitalOcean, Linode)	**45.2%**	9.7%	6.5%	3.2%	25.8%	3.2%	6.5%

**Table 22:** Teaching topics in the area of edge computing, 100% = 8.

To What Extent Do You Teach the Following Topics in the Area of Edge Computing?	Not at All	To a Small Extent	To Some Extent	To a Moderate Extent	To a Great Extent	To a Very Great Extent	No Answer
General concepts	0.0%	12.5%	12.5%	12.5%	25.0%	**37.5%**	0.0%
Privacy and security	12.5%	25.0%	12.5%	0.0%	**37.5%**	12.5%	0.0%
Scalability and reliability	0.0%	12.5%	25.0%	25.0%	12.5%	**25.0%**	0.0%
Speed and efficiency	0.0%	0.0%	25.0%	12.5%	37.5%	**25.0%**	0.0%
Applications	0.0%	0.0%	12.5%	25.0%	25.0%	**37.5%**	0.0%

**Table 23:** Technologies used in Edge Computing implementation, 100% = 8.

To What Extent Do You Teach the Following Technologies Used in Edge Computing Implementation?	Not at All	To a Small Extent	To Some Extent	To a Moderate Extent	To a Great Extent	To a Very Great Extent	No Answer
Mobile Edge Computing	12.5%	**37.5%**	12.5%	12.5%	0.0%	25.0%	0.0%
Fog computing	**25.0%**	**25.0%**	12.5%	0.0%	12.5%	12.5%	12.5%
Micro data centers	**25.0%**	12.5%	**25.0%**	12.5%	12.5%	12.5%	0.0%
Container technology	12.5%	**25.0%**	12.5%	12.5%	12.5%	12.5%	12.5%
Azure edge	**62.5%**	25.0%	0.0%	0.0%	0.0%	0.0%	12.5%
Service composition and service oriented computing	12.5%	12.5%	**25.0%**	**25.0%**	0.0%	**25.0%**	0.0%

**Table 24:** Algorithms/techniques used in Edge Computing implementation, 100% = 8.

To What Extent Do You Teach the Following Algorithms/Techniques Used in Edge Computing Implementation?	Not at All	To a Small Extent	To Some Extent	To a Moderate Extent	To a Great Extent	To a Very Great Extent	No Answer
Distributed computing	0.0%	12.5%	**37.5%**	0.0%	25.0%	25.0%	0.0%
Distributed storage	12.5%	12.5%	**37.5%**	0.0%	25.0%	12.5%	0.0%
Reliability and fault tolerance	12.5%	25.0%	**25.0%**	0.0%	**25.0%**	12.5%	0.0%
Containerization	12.5%	**37.5%**	0.0%	0.0%	12.5%	25.0%	12.5%
Energy efficiency	12.5%	**37.5%**	0.0%	12.5%	12.5%	25.0%	0.0%
Data replication	12.5%	25.0%	12.5%	0.0%	12.5%	**37.5%**	0.0%
Efficiently collecting, aggregating, and moving data	0.0%	**37.5%**	12.5%	0.0%	25.0%	25.0%	0.0%
Others	**37.5%**	0.0%	12.5%	25.0%	0.0%	12.5%	12.5%

**Table 25:** Students’ skills in Edge Computing, 100% = 8.

To What Extent Your Students…	Not at All	To a Small Extent	To Some Extent	To a Moderate Extent	To a Great Extent	To a Very Great Extent	No Answer
identify the challenges of edge computing?	0.0%	**37.5%**	**37.5%**	0.0%	25.0%	0.0%	0
design an edge computing architecture?	**25.0%**	**25.0%**	12.5%	12.5%	12.5%	12.5%	0
describe differences between edge, fog, cloud and pervasive computing?	12.5%	**25.0%**	**25.0%**	12.5%	**25.0%**	0.0%	0
implement SW solutions using edge-computing middlewares?	**25.0%**	**25.0%**	**25.0%**	12.5%	0.0%	12.5%	0
understand the strengths and weaknesses of an EC architecture?	0.0%	**37.5%**	12.5%	25.0%	12.5%	12.5%	0
develop an edge computing project?	**25.0%**	**25.0%**	12.5%	12.5%	0.0%	**25.0%**	0
read papers related to edge computing?	12.5%	**37.5%**	25.0%	0.0%	12.5%	12.5%	0
do data analytics in edge computing environments?	25.0%	**37.5%**	12.5%	12.5%	12.5%	0.0%	0

**Table 26:** Hardware/software EC enabling platforms used by students, 100% = 8.

To What Extent Do Your Students Use Hardware/Software Enabling Edge Computing Platforms?	Not at All	To a Small Extent	To Some Extent	To a Moderate Extent	To a Great Extent	To a Very Great Extent	No Answer
FPGAs	25.0%	**37.5%**	0.0%	12.5%	12.5%	0.0%	12.5%
Edge accelerators	25.0%	**50.0%**	0.0%	12.5%	0.0%	0.0%	12.5%
Azure IoT Edge	**75.0%**	12.5%	0.0%	0.0%	0.0%	0.0%	12.5%
AWS IoT Greengrass	**62.5%**	0.0%	12.5%	12.5%	0.0%	0.0%	12.5%
RTOS	**62.5%**	12.5%	12.5%	0.0%	0.0%	0.0%	12.5%

**Table 27:** Teaching applications of Edge Computing, 100% = 8.

To What Extent Do You Teach Edge Computing in the Following Applications?	Not at All	To a Small Extent	To Some Extent	To a Moderate Extent	To a Great Extent	To a Very Great Extent	No Answer
Autonomous machines	**50.0%**	0.0%	25.0%	12.5%	0.0%	12.5%	0.0%
Augmented reality	**62.5%**	25.0%	0.0%	0.0%	0.0%	12.5%	0.0%
Mobile agents (e.g., drones)	**62.5%**	0.0%	0.0%	25.0%	12.5%	0.0%	0.0%
Autonomous products	25.0%	25.0%	**37.5%**	0.0%	0.0%	12.5%	0.0%
Autonomy in energy networks	**62.5%**	25.0%	0.0%	12.5%	0.0%	0.0%	0.0%
Facial recognition algorithms	**62.5%**	12.5%	0.0%	0.0%	0.0%	25.0%	0.0%
Smart cities	25.0%	25.0%	25.0%	0.0%	0.0%	25.0%	0.0%
Industry 4.0	**37.5%**	25.0%	0.0%	12.5%	12.5%	12.5%	0.0%
Autonomous production planning system	**50.0%**	37.5%	0.0%	0.0%	12.5%	0.0%	0.0%

## Data Availability

Not applicable.
